# Modulation of experimental acute lung injury by exosomal miR-7704 from mesenchymal stromal cells acts through M2 macrophage polarization

**DOI:** 10.1016/j.omtn.2023.102102

**Published:** 2023-12-14

**Authors:** Wei-Ting Lin, Hao-Hsiang Wu, Chien-Wei Lee, Yu-Fan Chen, Lawrence Huang, Jennifer Hui-Chun Ho, Oscar Kuang-Sheng Lee

**Affiliations:** 1 Doctoral Degree Program of Translational Medicine, National Yang Ming Chiao Tung University and Academia Sinica, Taipei, Taiwan, R.O.C; 2 Institute of Clinical Medicine, National Yang Ming Chiao Tung University, Taipei, Taiwan, R.O.C; 3 Center for Translational Genomics & Regenerative Medicine Research, China Medical University Hospital, China Medical University, Taichung, Taiwan, R.O.C; 4 Department of Biomedical Engineering, China Medical University, Taichung, Taiwan, R.O.C; 5 Taipei American School, Taipei, Taiwan, R.O.C; 6 Department of Medical Research, Eye Center, China Medical University Hospital, China Medical University, Taichung, Taiwan, R.O.C; 7 Stem Cell Research Center, National Yang Ming Chiao Tung University, Taipei, Taiwan, R.O.C; 8 Department of Orthopedics, China Medical University Hospital, Taichung, Taiwan, R.O.C; 9 Department of Ophthalmology, China Medical University Hospital, Taichung, Taiwan, R.O.C

**Keywords:** MT: non-coding RNAs, IFN /TNF-stimulated MSCs, mesenchymal stromal cell, exosome, miR-7704, macrophage polarization, acute lung injury, MyD88/STAT1

## Abstract

Acute lung injury (ALI) is a life-threatening condition with limited treatment options. The pathogenesis of ALI involves macrophage-mediated disruption and subsequent repair of the alveolar barriers, which ultimately results in lung damage and regeneration, highlighting the pivotal role of macrophage polarization in ALI. Although exosomes derived from mesenchymal stromal cells have been established as influential modulators of macrophage polarization, the specific role of exosomal microRNAs (miRNAs) remains underexplored. This study aimed to elucidate the role of specific exosomal miRNAs in driving macrophage polarization, thereby providing a reference for developing novel therapeutic interventions for ALI. We found that miR-7704 is the most abundant and efficacious miRNA for promoting the switch to the M2 phenotype in macrophages. Mechanistically, we determined that miR-7704 stimulates M2 polarization by inhibiting the MyD88/STAT1 signaling pathway. Notably, intra-tracheal delivery of miR-7704 alone in a lipopolysaccharide-induced murine ALI model significantly drove M2 polarization in lung macrophages and remarkably restored pulmonary function, thus increasing survival. Our findings highlight miR-7704 as a valuable tool for treating ALI by driving the beneficial M2 polarization of macrophages. Our findings pave the way for deeper exploration into the therapeutic potential of exosomal miRNAs in inflammatory lung diseases.

## Graphical abstract

**Figure:**
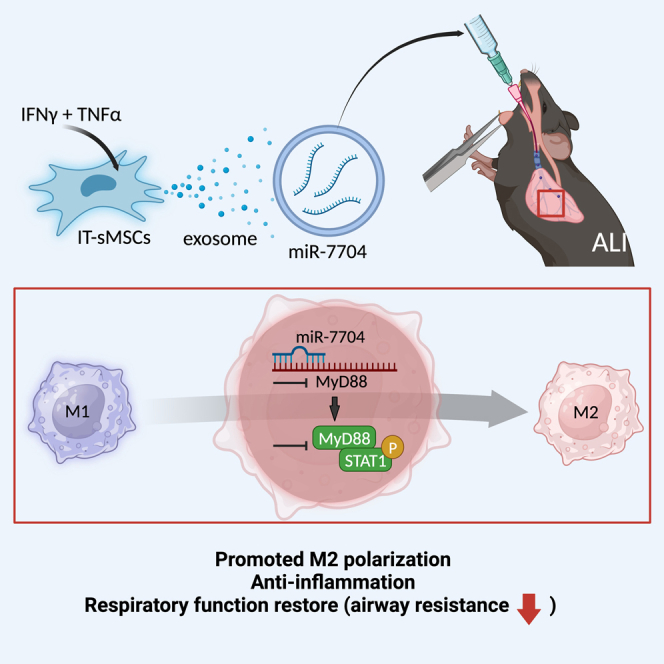

## Teaser

Lee, Ho, and colleagues investigated how exosomes from IFN-γ/TNF-α-stimulated MSCs can regulate macrophage polarization in treating ALI. They identify miR-7704 as a powerful candidate for shifting macrophages from M1 to M2 state, that effectively ameliorates ALI and restores lung function by directly targeting on the MyD88/STAT1 pathway.

## Introduction

Acute lung injury (ALI), a critical clinical respiratory condition, presents a considerable global health challenge with a notably high mortality rate, necessitating effective treatments.^1^^,^^2^^,^^3^ Commonly originating from underlying diseases such as sepsis, acute pneumonia, and acute pancreatitis, ALI triggers severe inflammation, leading to pathological damage in lung tissues. Clinical hallmarks include augmented pulmonary infiltration and extravascular fluid, decreased pulmonary compliance, and hypoxemia.^4^^,^^5^ Current treatment options include oxygen therapy and corticosteroids; however, the prognosis for lung function recovery remains poor. Selecting the appropriate treatment for ALI is challenging.^6^^,^^7^ Key players in the pathophysiological journey of ALI are macrophages, which play dichotomous roles in tissue regeneration, making them compelling therapeutic targets for regenerative medicine.^8^^,^^9^^,^^10^ Macrophages are generally classified into two major phenotypes, classically activated (M1) and alternative (M2) macrophages, functioning primarily in pro-inflammation and anti-inflammation activities, respectively. M1 macrophages are specifically involved in lung inflammation and tissue damage.^11^ Persistent M1 macrophage activation leads to excessive release of inflammatory cytokines, such as interferon γ (IFN-γ), tumor necrosis factor (TNF; previously known as TNF-α), interleukin 1 (IL-1*β*), and nitric oxide (NO), which is believed to contribute the most to the pathogenesis of ALI.^8^^,^^12^ Therefore, strategies to promote M2 macrophage polarization are emerging as potential approaches for the treatment of ALI treatments.^13^^,^^14^

In the quest for innovative therapeutic solutions, mesenchymal stromal cells (MSCs) have gained attention for the treatment of acute tissue damage due to their ability to modulate immune responses in an inflammatory environment, which occurs mostly through paracrine signals.^15^^,^^16^^,^^17^ Pre-stimulation MSCs with TNF and IFN-γ, so-called IFN-γ/TNF-stimulated MSCs (IT-sMSCs), is a widely accepted approach to enhance MSC potency for immunomodulation, particularly for inhibiting inflammation.^18^^,^^19^^,^^20^^,^^21^

MSCs serve as medicinal signaling cells by producing paracrine signals through exosomes.^22^ Exosomes are nano-sized extracellular vesicles less than 200 nm in diameter with a lipid bilayer structure. Exosomes are involved in intercellular communication and are, thus, considered potential therapeutics.^23^^,^^24^ Although exosomes are secreted by all cell types, MSC-derived exosomes have recently emerged as attractive cell-free therapeutics owing to their immunomodulatory effect.^25^^,^^26^^,^^27^^,^^28^ MSC-derived exosomes have been demonstrated to regulate gene expression in immune cells, including that regulating macrophage polarization.^29^^,^^30^^,^^31^ MicroRNAs (miRNAs) are abundant short non-coding RNAs with sequences of approximately 22 nucleotides that mediate gene silencing by targeting binding sites in the 3ʹ untranslated region (3ʹUTR) position of mRNAs.^32^^,^^33^ Exosomal miRNAs from MSCs have been proposed as a therapeutic approach for various diseases, including lung injury.^34^^,^^35^^,^^36^^,^^37^ However, information on key exosomal miRNAs and how they contribute to therapeutic outcomes of ALI remains limited.

Aiming to address this knowledge gap, in this study, we analyzed the role of specific exosomal miRNAs in ALI. By comparing the exosomal miRNA landscape between MSCs and IT-sMSCs, the response of the key exosomal miRNA miR-7704 to IFN-γ and TNF stimulation was identified. In addition, the mechanism underlying the amelioration of ALI by miR-7704 through regulating macrophage polarization was elucidated.

## Results

### Characterization of exosomes from MSCs and IT-sMSCs

**Figure 1:**
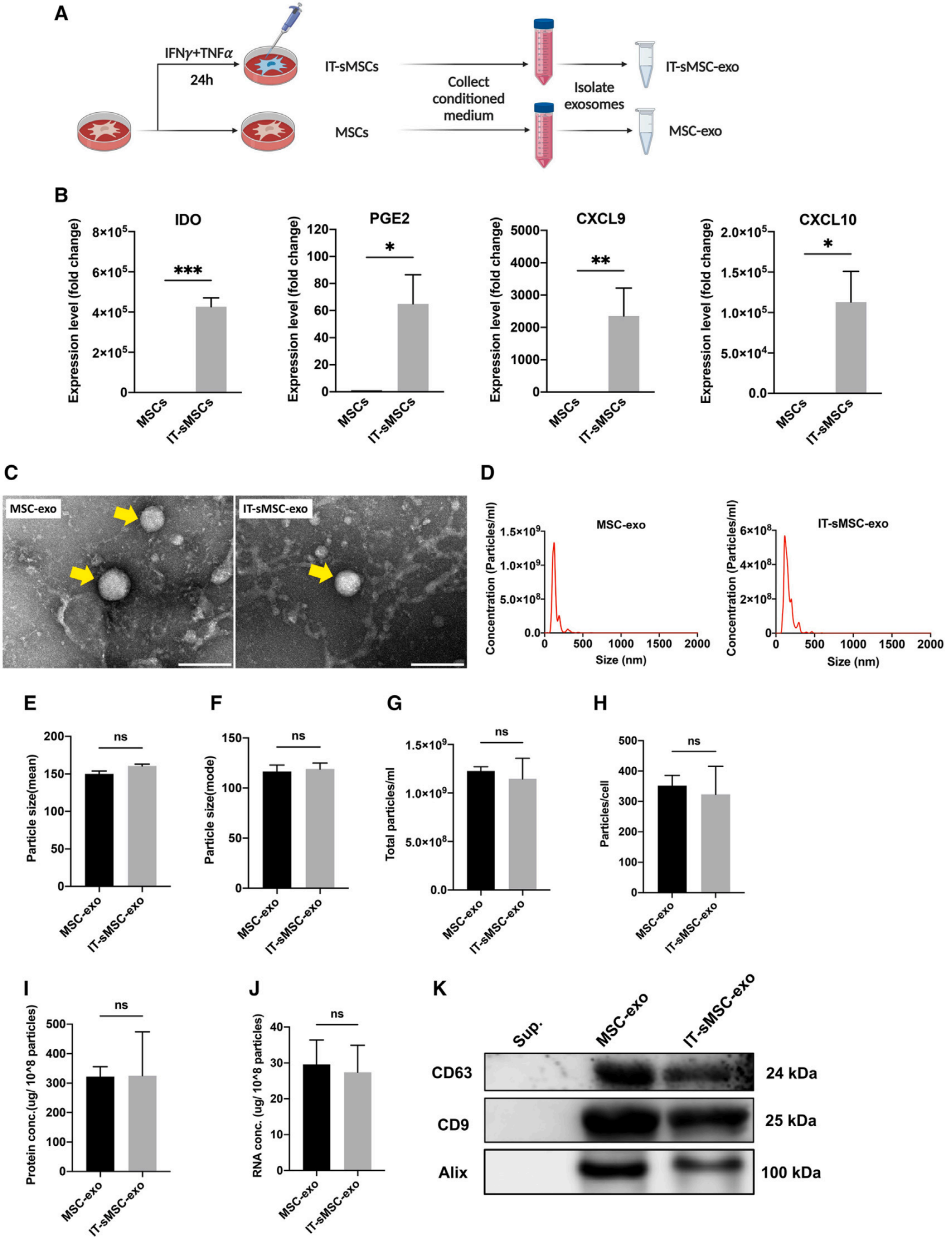
Characterization of MSC-exos and IFNγ/TNF-stimulated MSCs (A) Schematic showing the stimulating process and exosome isolation. MSCs were treated with TNF and IFN-γ for 24 h. (B) The expression of immuno-suppressive genes (IDO, PGE2, CXCL9, and CXCL10) in IT-sMSCs was measured using real-time qPCR (each group n = 6) normalized to the MSC group. (C) Transmission electron micrographs of MSC-exo and IT-sMSC-exo. The arrow indicates exosomes. Scale bar, 200 nm. (D–J) Nanoparticle tracking analysis revealed the size distribution (D), mean (E), and mode (F) diameter (each group n = 6). The quantification of different content in MSC-exo and IT-sMSC-exo from 50 mL of the CM: (G) total particles, (H) particles/cell, (I) protein concentration (μg/10^8^ cells), and (J) RNA concentration (μg/10^8^ cells) (each group n = 6). (K) Exosome markers CD63, CD9, and Alix were measured using western blotting analysis. Sup. = Supernatant. Results are shown as mean ± SD with data point. ∗p < 0.05, ∗∗p < 0.01, ∗∗∗p < 0.001; ns, not significant, Student’s two-tailed t test.

A combination of IFN-γ and TNF was treated in MSCs for 24 h (Figure 1A) to investigate the alternation of MSC immunoregulatory properties through cytokine stimulation (IT-sMSCs). As expected, MSCs provoked the expression of anti-inflammatory mediators following stimulation (Figure 1B) without changing MSC phenotype (Figure S1A) and multiple differentiation ability (Figures S1B–S1D). We then isolated exosomes from MSCs (MSC-exo) and IT-sMSCs (IT-sMSC-exo) separately. Both MSC-exo and IT-sMSC-exo exosomes showed typical morphology with a round shape under a transmission electronic microscope (Figure 1C) and an appropriate particle size of less than 200 nm through nanoparticle tracking analysis (Figure 1D). There was no significant difference in the particle size in terms of mean (Figure 1E) and mode (Figure 1F), respectively, between MSC-exo and IT-sMSC-exo. Moreover, there was a similar number of particles between MSC-exo and IT-sMSC-exo groups when normalized to the total volume of the conditioned medium (CM) (Figure 1G) or cell number (Figure 1H). When normalized to particle number, there was no difference in total protein concentration (Figure 1I) and RNA concentration (Figure 1J) between the MSC-exo and IT-sMSC-exo groups. Both MSC-exo and IT-sMSC-exo expressed strong exosome markers, such as CD63, CD9, and Alix (Figure 1K). The above findings suggest that IFN-γ and TNF stimulation on MSCs enhances anti-inflammatory ability without changing exosomal features, size, or the amount of total protein and total RNA in the exosome.

### IT-sMSC-exo is more potent than MSC-exo in inducing macrophage polarization

**Figure 2:**
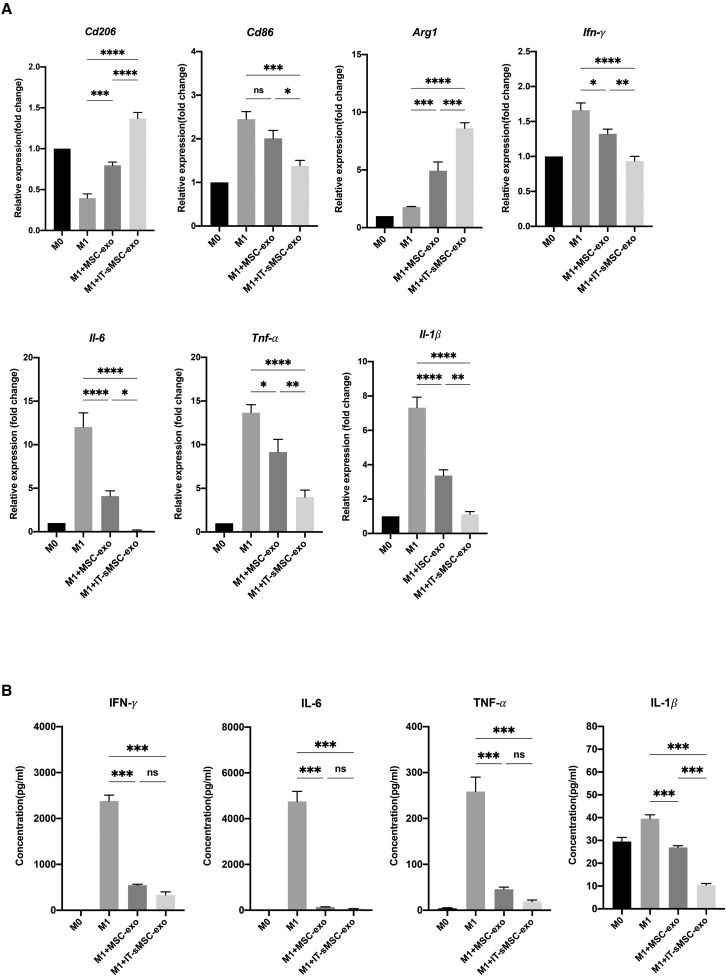
Effects on macrophage polarization of IT-sMSC-exos Twenty-four hours after the addition of MSC-exo and I/T-MSC-exo, macrophages were harvested and measured using real-time PCR (A) (*Cd206*, *Cd86*, *Arg1*, *Ifn-γ*, *Il-6*, *Tnf*, and *Il-1β*) (each group n = 6). (B) Inflammatory cytokine expression (IFN-γ, IL-6, TNF, and IL-1β) of macrophages was measured using ELISA (each group n = 6). Results are presented as mean ± SD. ∗p < 0.05, ∗∗p < 0.01, ∗∗∗p < 0.001; ns, not significant, one-way ANOVA.

The ability of MSC-exo and IT-sMSC-exo to cause macrophage polarization was compared. Macrophages, i.e., mouse bone marrow-derived macrophages (BMDMs), were activated from M0 to M1 by treating with lipopolysaccharide (LPS) and IFN-γ separately before incubation with MSC-exo or IT-sMSC-exo. Inflammatory cytokine gene expression (*IFN-γ*, *Il-6*, *TNF*, and *Il-1β*) (Figure 2A) in M1 macrophages and inflammatory cytokine secretion (IFN-γ, IL-6, TNF, and IL-1β) (Figure 2B) from M1 macrophages were significantly inhibited by MSC-exo and IT-sMSC-exo. The anti-inflammation potency of IT-sMSC-exo was superior to that of MSC-exo (Figure 2). Compared with MSC-exo, IT-sMSC-exo significantly downregulated the expression of CD86, the classical M1 phenotypic marker, and upregulated the expression of CD206, the M2 marker, in macrophages (Figure 2A). Based on these results, we infer that IT-sMSC-exo is more potent than MSC-exo as a biologic for modulating macrophage plasticity toward M2 phenotypic shifting.

### Exosomal miR-7704 in IT-sMSC-exo responsible for M2 macrophage polarization

**Figure 3:**
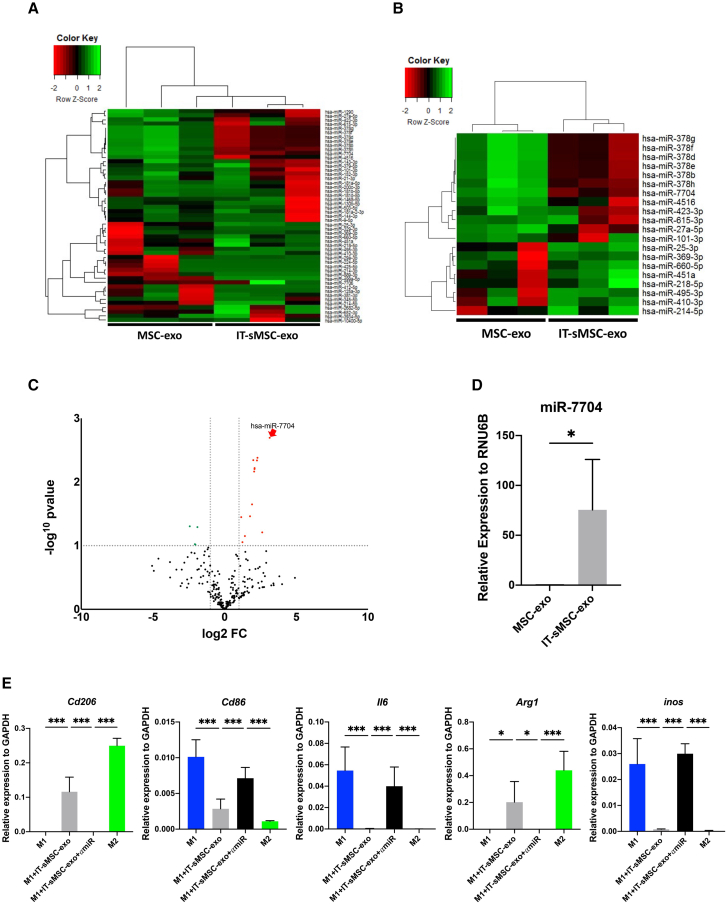
Differential miRNA profiles of IT-sMSC-exo and MSC-exo (A and B) Heatmap and © volcano plot histogram representative of exosomal miRNA differential expression from MSCs and IT-sMSCs. The abscissa represents log2 FC; ordinates represent significance (−log10 p value). A p value of <0.05 and log2FC of >1 were used to identify significantly differentially expressed miRNA; red dots represent significantly upregulated miRNA, and green dots represent significantly downregulated miRNA. miR-7704 is highlighted by the red arrow. (D) Exosomal miR-7704 expression from MSCs and IT-sMSCs was measured using qPCR (each group n = 6). (E) Effects of anti-miR7704 IT-sMSC-exo on M1 macrophages were analyzed using qPCR. Results are presented as mean ± SD. ∗p < 0.05, ∗∗p < 0.01, ∗∗∗p < 0.001; ns, not significant, Student’s two-tailed t test.

Exosomal miRNA sequencing was performed to elucidate miRNA profiling of MSC-exo and IT-sMSC-exo and to identify the major component contributing to IT-sMSC-exo-mediated anti-inflammation (Figures 3A and 3B). The top 10 upregulated miRNAs in IT-sMSC-exo, normalized by MSC-exo, are listed in Table S2. Upon transforming the sequencing data into a volcano plot, miR-7704 was identified as the most significantly upregulated miRNA in IT-sMSC-exo (Figure 3C). The upregulated exosomal miR-7704 expression in IT-sMSC-exo was confirmed through qPCR (Figure 3D). Using a loss-of-function assay in which an miR-7704 inhibitor was applied, the role of exosomal miR-7704 was investigated. M1 macrophages were incubated with IT-sMSC-exo plus anti-miR7704 (M1+IT-sMSC-exo+αmiR); the anti-inflammation effect and M2 polarization by IT-sMSC-exo were abolished (Figure 3E), suggesting that miR-7704 in IT-sMSC-exo plays a major role in reversing M1 to M2 polarization and inflammation inhibition.

### miR-7704 overexpressed in M1 macrophages leads to M2 polarization

**Figure 4:**
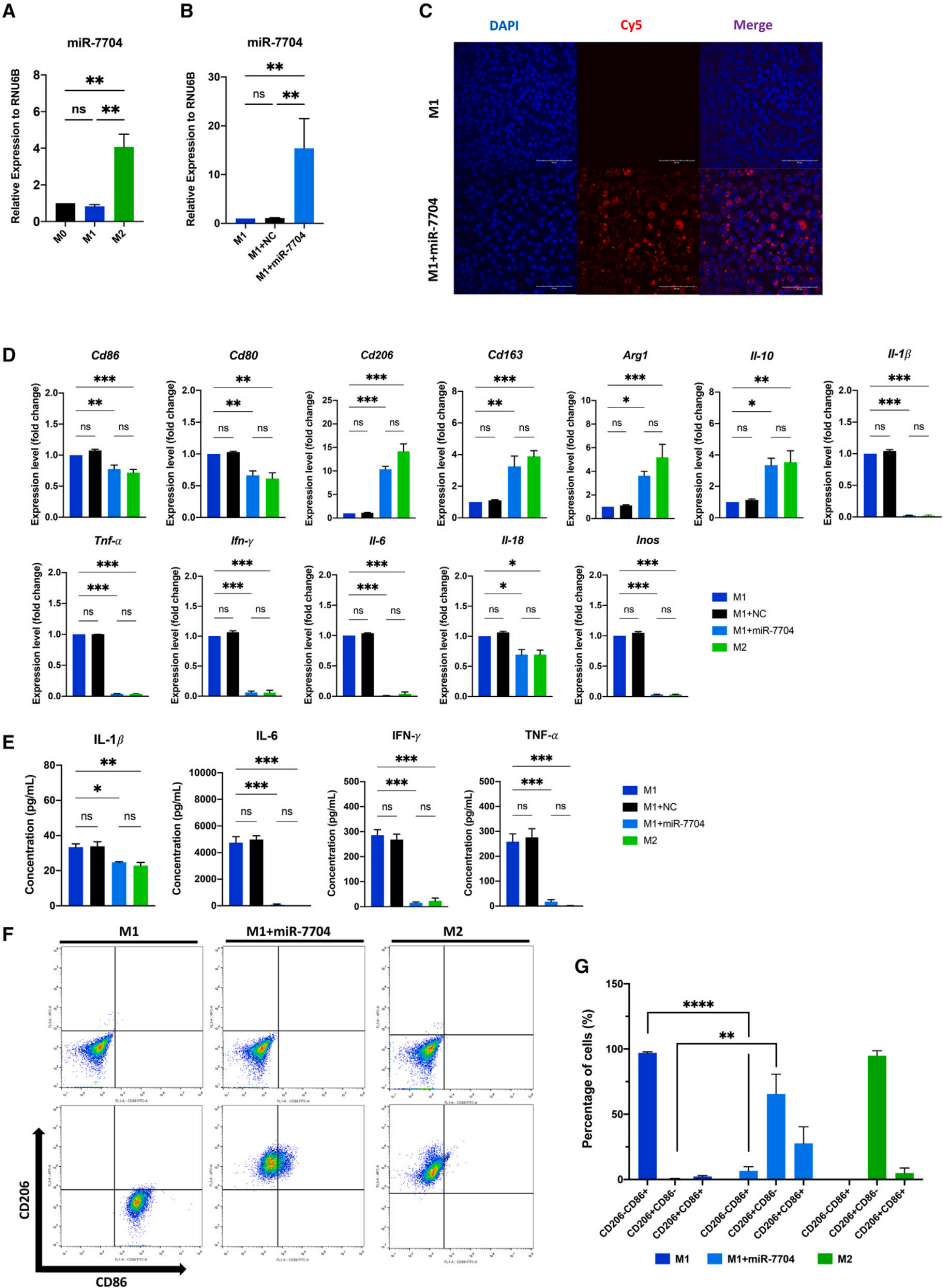
Analysis of effects on macrophage polarity by miR-7704 (A) miR-7704 expression in different phenotypes of macrophages (each group n = 6). The transfection efficiency of miR-7704 in M1 macrophages (M1+miR-7704) and NC (M1+NC) was analyzed using qPCR (B) and immunofluorescence staining conjugated with Cy5 (C) Scale bar, 90 μm (each group n = 6). (D) Macrophage surface makers (CD86 and CD206) and their quantified percentage (E) were analyzed using flow cytometry (each group n = 6). (F) Gene expression of M1, M1+NC, M1+miR-7704, and M2 macrophages. M1 marker genes (*Cd86*, *Cd80*, *Il-1β*, *TNF*, *IFN-γ*, *Il-6*, *Il-18*, and *inos*); M2 related genes (*Cd206*, *Cd163*, *Arg1*, and *Il-1*0) (each group n = 6). (G) Cytokine expression of M1, M1+NC, M1+miR-7704, and M2 through ELISA (IL1-β, IL-6, IFN-γ, and TNF; each group n = 6). Results are presented as mean ± SD. Statistical analyses were performed using one-way ANOVA. ∗p < 0.05, ∗∗p < 0.01, ∗∗∗p < 0.001; ns, not significant.

miR-7704 expression was significantly upregulated in IL-4-induced M2 macrophages (Figure 4A). Transfection of miR-7704 into M1 macrophages, confirmed through qPCR and immunofluorescence staining (Figures 4B and 4C), resulted in a direct effect of M2 shifting evidenced by phenotypic change. In an miR-7704-overexpressing M1 macrophage (M1+miR-7704), the gene expression of pro-inflammatory (*IL-1β, IFN-γ, TNF, Il-6, IL-18,* and *iNOS*) and M1 (*Cd80* and *Cd86*) markers was significantly downregulated, while M2 marker genes (*Cd206, Cd163, Arg-1,* and *Il-10*) were significantly increased (Figure 4D). The ability to produce pro-inflammatory cytokines (IL-1*β,* IL-6, IFN-γ, and TNF) was impaired in miR-7704-overexpressed M1 macrophages (Figure 4E). The phenotypic features of M1 macrophages measured using flow cytometry typically included CD86^+^CD206^−^ expression on the cell surface, while most M2 macrophages were CD86^−^CD206^+^-expressing cells. Overexpression of miR-7704 in M1 macrophages through transfection considerably increased the population of CD86^−^CD206^+^-expressing cells and reduced the proportion of CD86^+^CD206^−^-expressing cells (Figures 4F and 4G). Notably, regarding cytokine production ability and phenotyping, there were no differences between miR-7704-overexpressed M1 macrophages and M2 macrophages (Figures 4D–4G), suggesting that miR-7704 alone is potentially sufficient to convert M1 to M2 macrophages. These findings confirm the central role of miR-7704 in promoting M2 polarization.

### miR-7704 promotes M2 polarization via MyD88/STAT1 inhibition

**Figure 5:**
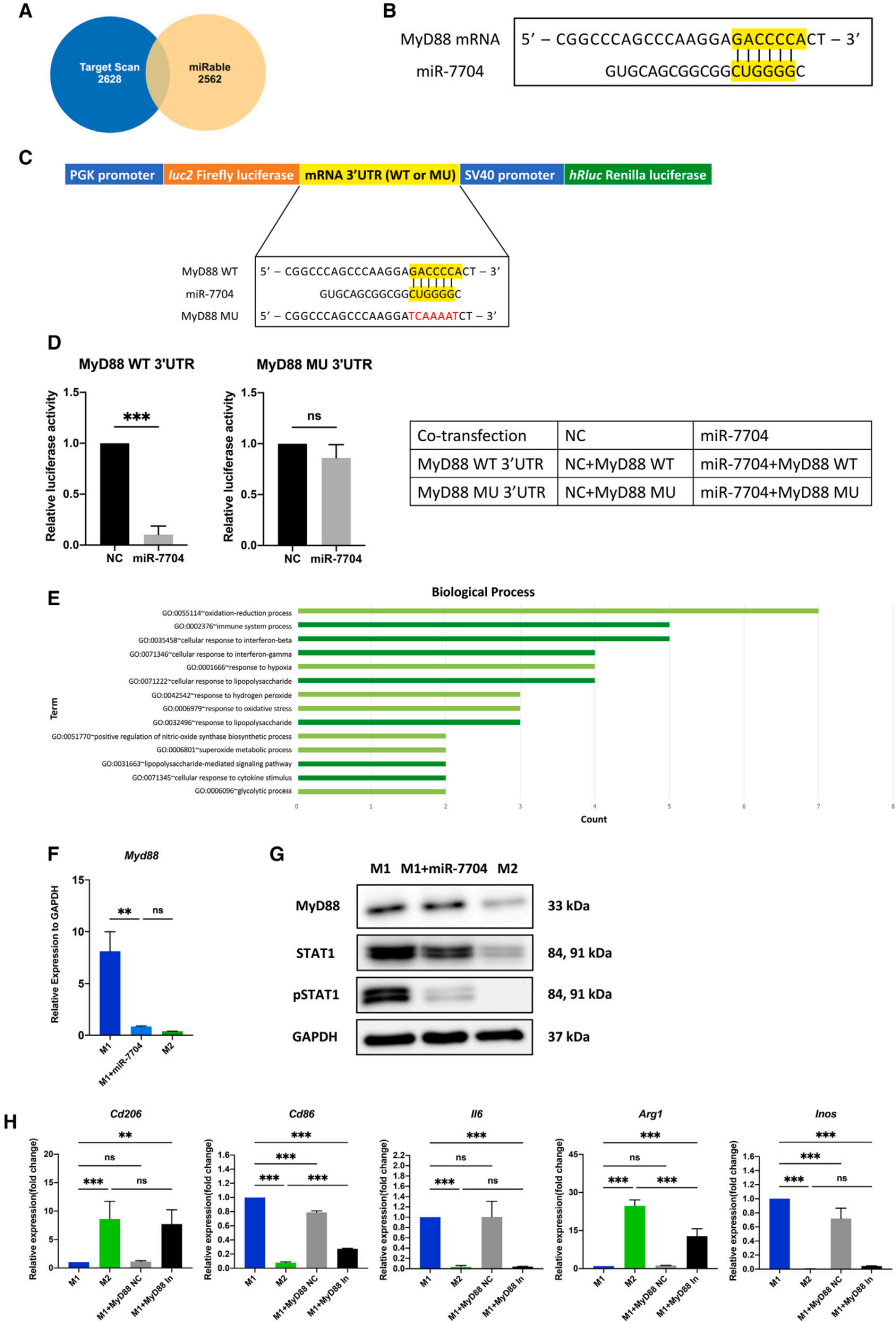
Validation of miR-7704 downstream signal pathway on promoting M2 polarization (A) Diagram of target prediction from two websites (Target Scan and miRabel). (B) Putative miR-7704 binding sites in 3ʹUTR of MyD88 mRNA. (C) Schematic representation of binding region between miR-7704 (or NC) and the 3′UTR of WT or MU MyD88 plasmid construction. (D) Transcriptional activity under co-transfection of plasmid and miRNA was determined through luciferase reporter assay in HEK293T cells. Relative luciferase activity was normalized to the Renilla:luciferase luminescence ratio (each group n = 6). (E) The bar chart represents Gene Ontology (GO) enrichment pathway from macrophage proteomics analysis. (F) Expression of Myd88 relative to GAPDH measured through qPCR (each group n = 6). (G) Immunoblotting of cell lysates from macrophages (MyD88, STAT1, and pSTAT1). (H) Effects of MyD88 inhibitor (MyD88 In) or NC on M1 macrophages (each group n = 6). Results are presented as mean ± SD. Statistical analyses were performed using one-way ANOVA. ∗p < 0.05, ∗∗p < 0.01, ∗∗∗p < 0.001; ns, not significant.

miRNAs downregulate mRNA by completely or partially targeting the binding site.^38^ Thus, the target mRNA of miR-7704, as well as the downstream signals regulated by miR-7704, are particularly important for understanding the therapeutic mode of action. Used in silico prediction (Target Scan^39^ and miRabel^40^) (Figure 5A) and proteomic analysis (Figure 5E; Table S5), a few potential pathways regulated by miR-7704 were proposed. Myeloid differentiation primary response gene-88 (*MyD88*) encodes one of the most critical regulators of M1 macrophage activation^11^^,^^29^ and was identified at the intersection of two prediction sites (Figure 5A). *MyD88* mRNA was predicted to be a binding target of miR-7704 (Figure 5B). To validate that miR-7704 targeted *MyD88*, we co-transfected miR-7704 with plasmid-conjugated 3′UTR of wild-type (WT)- or mutant type (MU)-MyD88 mRNA to HEK293 cells (Figure 5C). The luciferase activity of WT-MyD88 mRNA was significantly inhibited, while that of MU-MyD88 was not inhibited (Figure 5D), supporting that MyD88 is a direct target of miR-7704. Moreover, qPCR and Western blot analyses showed that miR-7704-overexpressed M1 macrophages reduced the MyD88 expression level equal to the expression level in M2 macrophages (Figures 5F and 5G). Proteomic analysis was performed on M1 macrophages and miR-7704-overexpressed M1 macrophages to investigate the MyD88 downstream signaling pathway regulated by miR-7704. It was revealed that STAT1-mediated cellular/immune response to IFNs, LPS, cytokines, and oxidative stress-induced inflammatory response in M1 macrophages was significantly downregulated by miR-7704 via Gene Ontology analysis (Figure 5E; Table S5). In addition, STAT1 and phosphorylated STAT1 were significantly reduced in M1 macrophages after miR-7704 transfection (Figure 5G). MyD88 has been reported to form complexes with STAT1 in macrophages.^41^^,^^42^ To further elucidate the impact of MyD88 inhibition on M2 polarization, M1 macrophages were treated with a MyD88 inhibitor or a negative control (NC). With the MyD88 blockade, similar to the effect of miR-7704 overexpression, M1 macrophages switched to the M2 phenotype (Figure 5H). These findings support the notion that miR-7704 affects the phenotypic alternation of macrophages toward M2 polarization by inhibiting MyD88/STAT1.

### miR-7704 ameliorated LPS-induced ALI

**Figure 6:**
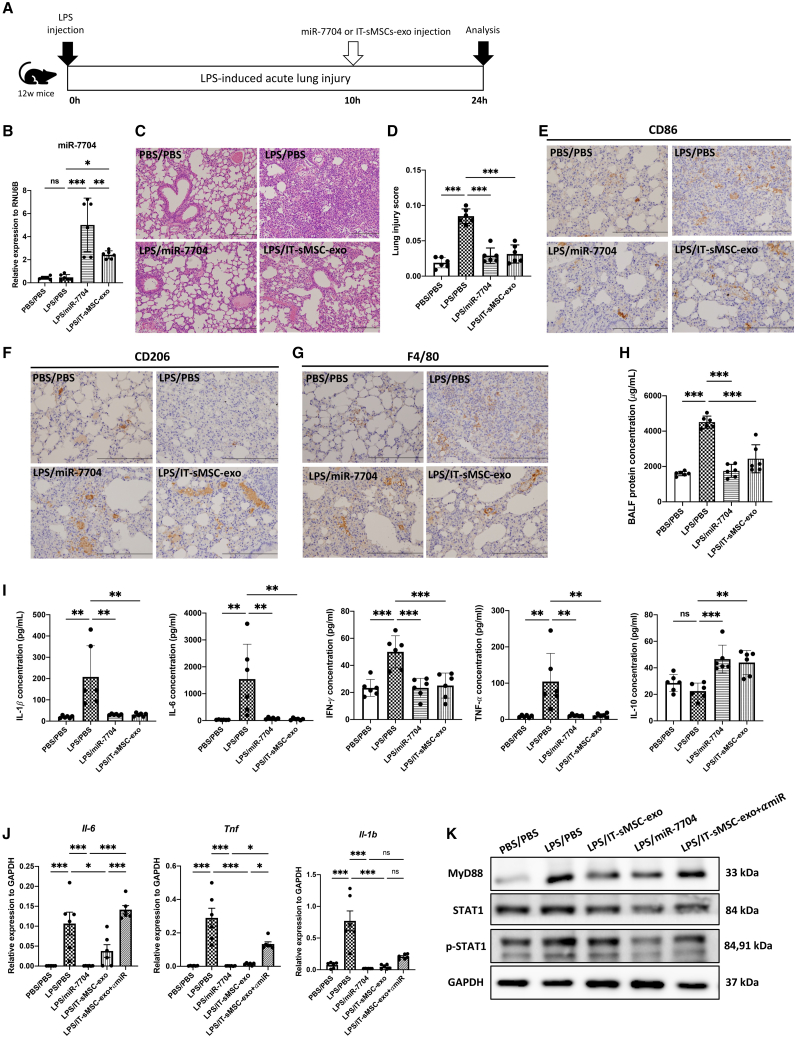
Amelioration of LPS-induced ALI by miR-7704 (A) Schematic showing the experimental design of ALI, control (PBS/PBS), LPS (LPS/PBS), miR-7704 (LPS/miR-7704), IT-sMSC-exo (LPS/IT-sMSC-exo), IT-sMSC-exo plus anti-miR-7704 (LPS/IT-sMSC-exo+αmiR) administration, and analysis (each group n = 6). (B) miR-7704 expression in different groups (each group n = 6). (H) BALF protein concentration in different groups (each group n = 6). (C) Representative H&E staining in different groups. Scale bar, 200 μm. (D) Lung injury score in different groups. Representative IHC staining for CD86 (E), CD206 (F), and F4/80 (G) expression in different groups under 200× magnification. Brown: positively stained cells. Scale bar, 200 μm. (H) Protein concentration of BALF in different groups. (I) Cytokine expression of BALF measured using ELISA (IL1-β, IL-6, IFN-γ, TNF, and IL-10). (J) Inflammatory-related gene expression of lung tissue through real-time PCR in different groups (each group n = 6). (K) Immunoblotting of lung tissue protein (MyD88, STAT1, and pSTAT1). Results are presented as mean ± SD. Statistical analyses were performed using one-way ANOVA. ∗p < 0.05, ∗∗p < 0.01, ∗∗∗p < 0.001; ns, not significant.

To validate the therapeutic effect of miR-7704 and IT-sMSC-exo for treating ALI, LPS was intratracheally injected into a 12-week-old mouse using a micro sprayer aerosolizer for disease model establishment. miR-7704 (labeled LPS/miR-7704 in Figure 6) or IT-sMSC-exo (labeled LPS/IT-sMSC-exo in Figure 6) was administered intratracheally 10 h after LPS induction (Figure 6A). The expression of miR-7704 in lung tissues was increased in the miR-7704-treated group (Figure 6B). A severe cellular infiltration (Figure 6C), evident in the lungs of mice with LPS-induced ALI, and the lung injury score^43^ (Figure 6D) were significantly reduced upon treatment with miR-7704 or IT-sMSC-exo. Immunohistochemical staining revealed more CD86-positive cells and fewer CD206-positive cells in the lung tissues with ALI (LPS/PBS) than in the normal lung tissues (PBS/PBS), indicating that M1 macrophages were dominant in the injured lung (Figures 6E and 6F). Treatment with miR-7704 (LPS/miR-7704) or IT-sMSC-exo (LPS/IT-sMSC-exo) decreased CD86-positive cells and increased CD206-positive cells compared with those in lung tissues with ALI (LPS/PBS), indicating that both miR-7704 and IT-sMSC-exo increased the M2 macrophage population in the injured lung (Figures 6E and 6F). Interestingly, changes in the number of macrophages were not evident following staining with macrophage marker F4/80 (Figure 6G), suggesting that inflammatory inhibition by miR-7704 and IT-sMSC-exo in ALI is more relevant to the regulation of macrophage plasticity, rather than the elimination of macrophages. Previous research has shown that continuous, severe inflammation triggers pulmonary fibrotic change.^29^ We observed that intensive lung inflammation induced fibrotic signals, i.e., collagen deposition stained with Masson trichome in the lung tissue (Figure S2A), and their quantified intensity (Figure S2B) was diminished upon treatment with miR-7704. These findings suggest that miR-7704 alleviates inflammatory signals and prevents lung fibrosis.

Bronchoalveolar lavage (BAL) is a common experimental method used to assess cellular and non-cellular components in the lungs.^44^ In the context of pathophysiology, ALI leads to the collapse of the alveolar-capillary membrane barrier, resulting in increased lung permeability. In this study, we collected BAL fluid (BALF) from ALI mice to investigate their inflammatory responses and disease progression. The BALF protein concentration in ALI was significantly reduced when mice were injected with miR-7704 or IT-sMSC-exo (Figure 6H). Both miR-7704 and IT-sMSC-exo treatments led to a reduction in the levels of inflammatory cytokines in BALF, including IL-1β, IL-6, IFN-γ, and TNF in ALI (Figure 6I). Furthermore, the level of the anti-inflammatory cytokine IL-10 significantly increased following miR-7704 and IT-sMSC-exo treatments (Figure 6I). Flow cytometry analysis was conducted to examine the cellular components in BALF, revealing similar trends in macrophage phenotypic changes in ALI mice (Figure S3). There were no significant differences in CD11b expression, which serves as a pan marker for macrophages among BALF-derived cells (Figures S3A–S3D). In the LPS-induced ALI mice (LPS/PBS), CD86-positive macrophages were more abundant, indicating an enhanced inflammatory response. However, this effect was mitigated following injection with miR-7704 (LPS/miR-7704) or IT-sMSC-exo (LPS/IT-sMSC-exo) (Figures S3E–S3H). Notably, CD206-positive M2 macrophages showed a significant increase in the miR-7704 and IT-sMSC-exo treatment groups (Figures S3E–S3H). Importantly, the amelioration of inflammatory signaling was reversed in mice treated with IT-sMSC-exo plus anti-miR-7704 (IT-sMSC-exo+αmiR) (Figure 6J). In addition to the anti-inflammatory perturbation, hematoxylin and eosin (H&E) staining revealed severe cellular infiltration in the lung tissue (Figure S2C, labeled as LPS/IT-sMSC-exo+αmiR). This was accompanied by an increase in CD86 expression (Figure S2D, labeled as LPS/IT-sMSC-exo+αmiR) and a decrease in CD206 expression (Figure S2E, labeled as LPS/IT-sMSC-exo+αmiR) in the lung tissue of mice in the IT-sMSC-exo+αmiR group, suggesting the crucial role of miR-7704 in ALI treatment. miR-7704 was found to alter the phenotypic changes in macrophages toward M2 polarization by inhibiting the MyD88/STAT1 signaling pathway *in vitro* (Figure 5). Subsequently, we analyzed the expression of MyD88 and STAT1 in the lung tissue of ALI mice. The expression of MyD88, STAT1, and phosphorylated STAT1 was downregulated in miR-7704- (LPS/miR-7704) and IT-sMSC-exo-treated (LPS/IT-sMSC-exo) groups, but not in ALI mice injected with IT-sMSC-exo plus anti-miR-7704 (Figure 6K). The results confirmed that miR-7704 promotes M2 polarization and anti-inflammation by regulating the MyD88/STAT1 axis in ALI mice.

According to our sequencing data, the miR-378 family was highly enriched in IT-sMSC-exo (Table S2). ALI mice that received miR-378 (LPS/miR-378) injection exhibited a reduction in inflammatory signals (Figure S2C, labeled as LPS/miR-378). However, it is worth noting that the ameliorating effects of miR-7704 were significantly more pronounced than those of miR-378 (Figure S2F). Notably, we did not observe any phenotypic changes in macrophages when mice were injected with miR-378; there were no discernible differences in the expression of CD86 and CD206 in the lungs of ALI mice (Figures S2D and S2E, labeled as LPS/miR-378). These results indicate the dominant role of exosomal miR-7704 in LPS-induced ALI in mice.

**Figure 7:**
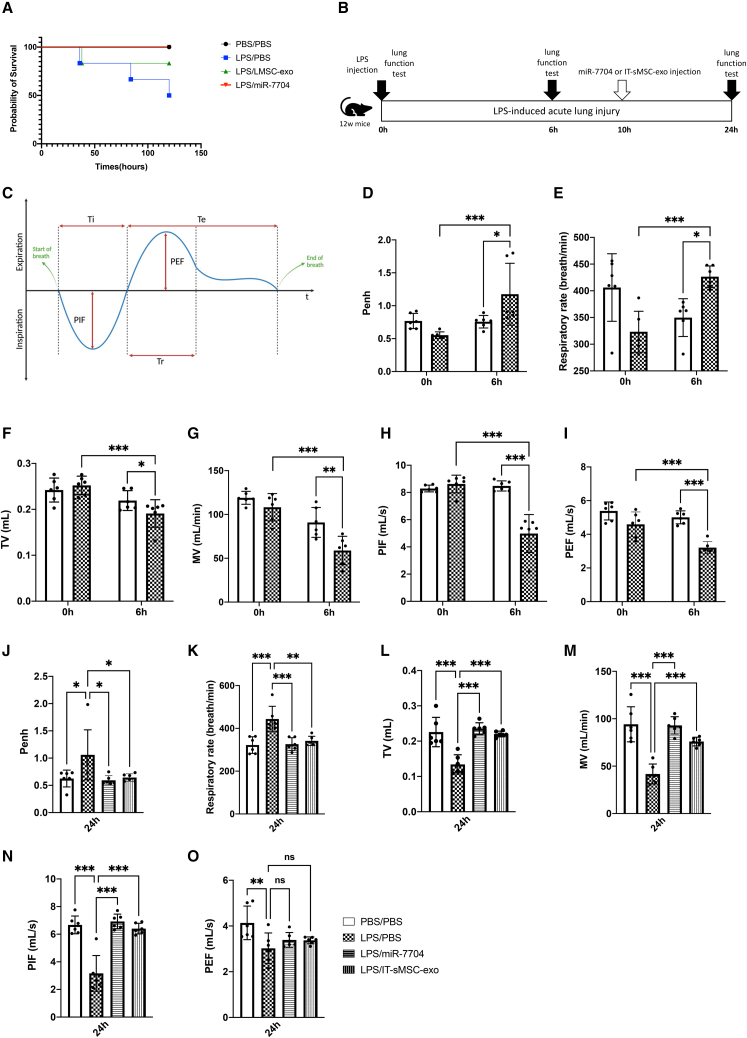
miR-7704 restored respiratory function from LPS-induced ALI (A) Survival curve of LPS-induced mice following miR-7704 and IT-sMSC-exo injection. Log rank test, p = 0.06. (B) Schematic showing the experimental design of ALI and lung function test on baseline (0 h), 6 h, and 24 h. (C) Lung function in mice measured through whole-body plethysmography. Respiratory parameters: P_enh_, respiratory rate, TV, MV, PIF, and PEF. (D–I) Lung function test at 0 h and 6 h. P_enh_ (D), respiratory rate (E), TV (F), MV (G), PIF (H), and PEF (I) (each group n = 6). (J–O) Lung function test at 24 h. P_enh_ (J), respiratory rate (K), TV (L), MV (M), PIF (N), and PEF (O) (each group n = 6). Results are presented as mean ± SD. Statistical analyses were performed using two-way ANOVA. ∗p < 0.05, ∗∗p < 0.01, ∗∗∗p < 0.001; ns, not significant.

Our findings revealed that the administration of miR-7704 or IT-sMSC-exo significantly reduced the mortality rate. The survival rate at 24 h after treatment was significantly higher in the miR-7704 (LPS/miR-7704) or IT-sMSC-exo (LPS/IT-sMSC-exo) treatment groups compared with that in the LPS-induced groups (LPS/PBS) (Figure 7A). These results demonstrate that intra-tracheal delivery of miR-7704 effectively improves survival in ALI.

To study whether miR-7704 or IT-sMSC-exo rescues lung function after lung injury, respiratory parameters were measured at baseline before LPS (or PBS) injection (0 h), at 6 h after injection of LPS (or PBS), and 24 h after LPS (or PBS) injection (14 h after miR-7704 or IT-sMSC-exo treatment) (Figure 7B). Respiratory parameters within the cycle of breath, including respiratory rate (breath per minute) and peak expiratory flow (PEF), tidal volume (TV), minute volume (MV), and peak inspiratory flow (PIF) of the mice were measured through Buxco Small Animal Whole Body Plethysmography^45^ (Figure 7C). Airway resistance was estimated with enhanced pause (P_enh_).^46^^,^^47^^,^^48^^,^^49^ A decrease in TV, MV, and PIF commonly occurs along with pulmonary damage, while an increase in respiratory rate (shortness of breath) and airway resistance (P_enh_) accompany ALI.^46^^,^^47^^,^^48^^,^^49^

In the current study, we demonstrated that 6 h after PBS injection, compared with baseline prior to injection, the PBS injection group (PBS/PBS) was relatively tolerated by the animals without affecting P_enh_ (Figure 7D), respiratory rate (Figure 7E), TV (Figure 7F), MV (Figure 7G), PIF (Figure 7H), or PEF (Figure 7I). However, after LPS injection for 6 h (LPS/PBS), lung function impairments were evidenced by the significantly increasing P_enh_ (Figure 7D) and respiratory rate (Figure 7E) and markedly decreasing TV (Figure 7F), MV (Figure 7G), PIF (Figure 7H), and PEF (Figure 7I). With miR-7704 or IT-sMSC-exo treatment for 14 h (24 h after LPS injection), LPS-induced airway resistance (P_enh_) (Figure 7J) and shortness of breath (Figure 7K) subsided and were well controlled by miR-7704 or IT-sMSC-exo. In addition, the decrease in TV (Figure 7L), MV (Figure 7M), and PIF (Figure 7N) caused by LPS-induced lung tissue damage could be recovered by treating with miR-7704 or IT-sMSC-exo. At 24 h, miR-7704 or IT-sMSC-exo treatment for 14 h (24 h after LPS injection) did not affect PEF (Figure 7O). These findings suggest that miR-7704 and IT-sMSC-exo not only ameliorate lung inflammation, but also restore pulmonary functions and repair tissue damage in LPS-induced ALI.

## Discussion

To the best of our knowledge, this report is the first demonstrating that miR-7704, a potential therapeutic miRNA, exhibits substantial enrichment within exosomes derived from IT-sMSCs. These exosomes effectively induce the polarization of macrophages from the M1 to M2 the phenotype, contributing to the amelioration of ALI and the facilitation of pulmonary function recovery. Mechanistically, miR-7704 promoted M2 macrophage polarization by directly binding to MyD88 and subsequently inhibiting the MyD88/STAT1 signaling pathway. MSCs treated with IFN-γ and TNF provide a therapeutic strategy for ALI treatment by enriching miR-7704 in the exosomes. MSCs can deliver versatile therapeutic miRNAs via exosomes. However, the mechanism of each exosomal miRNA participating in inflammation processes has not been fully studied. Through sequencing of exosomal miRNA derived from MSCs, miR-7704 was identified as the top differential miRNA upregulated after MSC stimulation by TNF and IFN-γ. miR-7704 plays a crucial role in regulating macrophage polarization, which results in inflammation inhibition, survival improvement, and pulmonary function recovery.

miR-7704 originates from the exon region of chromosome 2 (from 5ʹ-176,188,843 to 176,188,901-3ʹ). Notably, *HOXD1* (Homeobox D1), a protein-coding gene, specifically serves as the host gene for miR-7704 (Figure S4).^50^ miR-7704 is located in the promoter region, as indicated by the blue box in Figure S4. In our study, MSCs were pre-treated with TNF and IFN-γ to enhance their immunomodulatory capability. This process resulted in a significant enrichment of exosomal miR-7704 in IT-sMSC-exo. The enhancement of miR-7704 expression in IT-sMSC-exo due to TNF and IFN-γ exposure may involve direct or indirect regulatory interactions with *HOXD1*. The selective sorting of miRNAs into exosomes is a complex process that remains incompletely understood. Current hypotheses propose the involvement of multiple factors, including specific sequences and motifs within the miRNA sequences, as well as the presence of particular proteins.^51^^,^^52^ Notably, studies suggest that the endosomal sorting complexes required for transport machinery, along with other proteins, may contribute to the intricate process of sorting miRNAs into exosomes.^23^ However, there are no well-documented or widely reported direct interactions between the promoter region of *HOXD1* and these cytokines. To explore this potential regulatory interaction, approaches such as chromatin immunoprecipitation and luciferase reporter assay could help to reveal these interactions.

Increasing evidence suggests that macrophage polarization is a crucial determinant in most tissue injuries mediated by extensive inflammation. Macrophage polarization results in diverse immune responses mediated by pro- or anti-inflammatory cytokines, which exert systemic effects on other immune cells. In this study, we observed the function of miR-7704 in polarizing macrophages from M1 to M2 phenotypes. Classic M1 macrophages expressed CD86^+^CD206^−^, while M2 macrophages typically expressed CD86^−^CD206^+^. After miR-7704 transfection to M1 macrophages, most M1 macrophages showed a significant phenotypic change and reverted to M2-like macrophages (∼65.61%). However, we observed a population of cells expressing both CD86^+^CD206^+^ (27.78%) (Figures 4F and 4G), supporting that the phenotypic change in macrophages is dynamic. Macrophages may appear simultaneously in intermediate and steady states rather than only the two canonical populations.^53^

The complexity of signaling pathways governing phenotypic alterations has not been fully investigated. Proteomic analyses revealed that with IFN-γ and LPS stimulation, the levels of inflammatory proteins in the miR-7704-overexpressed M1 macrophages were significantly lower than those in M1 macrophages, and STAT1 was the most significantly impacted by miR-7704 (Table S5; Figure 5E). Downregulation of proteins involved in oxidative and glycolytic processes, such as NOS2 (inducible NO synthase [iNOS]) and SOD2, was observed in miR-7704-transfected M1 macrophages (Table S5; Figure 5E). Macrophages with high levels of iNOS and SOD2 expression are strongly correlated with the M1 phenotype and undergo metabolic reprogramming toward glycolysis and fatty acid synthesis, resulting in reactive oxygen species formation and interruption of the Krebs cycle.^54^

miR-7704 significantly inhibited M1 activity by directly binding to MyD88 and inhibiting the MyD88/STAT signaling pathway (Figure 5). The STAT family plays an important role in macrophage polarization; for instance, STAT1 and STAT3 are involved in M1 activity, while STAT6 is involved in M2 activity. Inhibition of MyD88 downregulates STAT1 and its phosphorylation. The balance of STAT family members determines the M1/M2 status.^11^^,^^53^ STAT1 is widely recognized as one of the pivotal players in inflammatory responses.^55^ The phosphorylation of STAT1 typically follows activation of Toll-like receptor 4 (TLR4) upon binding with LPS. This signal transduction depends on upstream MyD88 activation.^41^^,^^42^^,^^55^^,^^56^ MyD88 regulates STAT1 phosphorylation through the activation of the p38 MAPK pathway and augments the expression of endogenous genes such as *IFN*-*γ*.^57^ The mechanism of action was also observed in our ALI mouse model (Figure 6). Numerous studies have highlighted the critical role of MyD88 in alveolar inflammation. MyD88 serves as an important adaptor protein in activating TLRs, such as TLR4 activated by LPS.^58^ Mice lacking MyD88 molecules exhibit a notable reduction in pro-inflammatory responses and lung protein leakage.^58^^,^^59^ Additionally, specific knockout of MyD88 in macrophages blocks LPS-induced inflammation in mice.^60^ In addition to MyD88, many putative miR-7704-binding mRNAs were predicted by using Target Scan and miRabel. Whether these mRNAs regulate M1/M2 macrophage polarization, other than through the MyD88/STAT1 signaling pathway, is currently being investigated in our laboratory.

ALI typically goes through three stages of tissue injury.^4^ The initial stage, the exudative phase, is associated with inflammatory responses that cause alveolar damage. The following stage, the proliferative phase, is mainly characterized by the repair of alveolar and epithelial cells. The third stage, the fibrotic phase, occurs in a certain subset of patients who manifest pulmonary fibrosis. In the exudative phase, macrophages are recruited to the lungs as the first line of defense against pathogens in the respiratory tract. Immune cells, such as neutrophils or T cells, are recruited and activated in the presence of pro-inflammatory cytokines secreted by macrophages, and the release of other factors, such as oxidants, leads to the progression of pulmonary injury. In a macrophage-depleted mouse, LPS-induced lung injury was not evident, indicating that macrophages are crucial for ALI pathological progression.^8^ We particularly detected pulmonary function in ALI after miR-7704 and IT-sMSC-exo treatment to gain insights into their clinical importance (Figure 7). In the present study, treatment with IT-sMSC-exo and miR-7704 effectively reduced pro-inflammatory signals while promoting M2 macrophage activation (Figures 6C–6F, 6H, 6I, and S3) accompanied by pulmonary function recovery (Figure 7) and ameliorating collagen deposition (Figures S2A and S2B). This result indicated that exosomal miR-7704 is a potential therapeutic for abrogating ALI-induced pulmonary fibrosis.

In addition to exosomal miRNA, various other factors play a crucial role in MSC-based therapy for diseases like ALI, sepsis, and end-organ injuries, which have been reported in the literature. For instance, MSCs have been shown to attenuate sepsis and ALI/ARDS through systemic anti-inflammatory responses, partially achieved via the secretion of IL-10.^61^^,^^62^ It is worth noting that a single molecule or pathway alone is not likely to provide the full protective effect of MSC-based therapy. Instead, a wide range of immune and metabolic modulatory networks that target different cell types and organs coordinate to achieve therapeutic effects.^63^

miRNA-based biologics have been highlighted as potential therapeutics for the treatment of lung diseases.^35^^,^^36^^,^^64^ For example, miR-377 and miR-384 released by MSC exosomes were reported to ameliorate LPS-induced ALI by increasing macrophage autophagy.^65^^,^^66^ In our study, several miRNAs other than miR-7704 were highly enriched in IT-sMSC-exo, including the miR-378 family (miR-378b, miR-378d, miR-378e, miR-378f, miR-378g, miR-378h, and miR-378i) (Table S2). The miR-378 family has been reported to exert protective effects against liver fibrosis, liver injury, and kidney diseases; promote wound healing; and ameliorate nerve injury.^67^^,^^68^^,^^69^^,^^70^^,^^71^ In the present study, miR-378 demonstrated partial amelioration of ALI, although its effects were not as significant as those observed with miR-7704 (Figure S2). As a result, further investigation is warranted to explore the therapeutic potential of IT-sMSC-exos, particularly those containing the miR-378 family, for multiple indications in the future.

An intratracheal injection was applied for the administration of LPS, IT-sMSC-exo, and miR-7704. Among intratracheal, intravenous, intraperitoneal, and intranasal administration of rats, intratracheal instillation of liquid miRNA provides the most efficient drug delivery to deep lung tissue.^72^ Delivery of drugs through the respiratory tract not only increases drug concentration in the lung, but also reduces off-target effects. However, miRNA in the respiratory tract may be cleared rapidly by intrinsic pulmonary clearance mechanisms; to overcome the short half-life of miRNA in the airway, strategies such as the use of lipids, polymer nanoparticles, or exosomes as carriers for miRNA delivery to the lungs would prolong the half-life and increase therapeutic efficiency of miRNA in lung tissues.

In conclusion, our results underscore the therapeutic potential of exosomal miR-7704 from IT-sMSCs in the context of ALI. This opens a new avenue for the development of miRNA-based therapeutics for lung diseases and potentially other inflammatory conditions. Future studies are needed to further elucidate the mechanistic pathways underlying these observed effects and to optimize the delivery of these potential therapeutic agents.

## Materials and methods

### LPS-induced ALI mouse model

Twelve-week-old male C57BL/6JNarl mice were obtained from the National Laboratory Animal Center (NAR Labs) and maintained under specific pathogen-free conditions with controlled temperature and humidity at the National Yang Ming Chiao Tung University Laboratory Animal Center, Taiwan. Animal experiments were approved by the National Yang Ming Chiao Tung University Animal Care and Use Committee (IACUC NO. 1110705). After 2 weeks of acclimatization, the mice were randomly divided into the following groups: (1) PBS/PBS, (2) LPS/PBS, (3) IT-sMSC-exos (LPS/IT-sMSC-exo), (4) miR-7704 (LPS/miR-7704), (5) IT-sMSC-exo plus anti-miR-7704 (LPS/IT-sMSC-exo+αmiR), and (6) miR-378 (LPS/miR-378) (n = 6 per group). Mice were subjected to 20 mg/kg LPS treatment (LPS from *Escherichia coli* O111:B4, L2630, Sigma) via intratracheal injection to generate an ALI model. Ten hours after LPS injury, 200 nM miR-7704, 200 nM miR-378, 50 μg IT-sMSC-exo, or 50 μg IT-sMSC-exo plus 200 nM anti-miR was administered intratracheally and slowly delivered into the lungs using a micro-sprayer aerosolizer (Shanghai Yuyan Instruments Co., Ltd., YAN30012). Mice were euthanized through isoflurane inhalation overdose 24 h after LPS injury. The right lung tissues were collected for analysis and stored at −80°C for RNA extraction and protein isolation.

### BALF analysis

BALF was collected from the mice by inserting a 26G needle into the trachea and instilling 1 mL PBS. In brief, PBS was gently injected and aspirated from the lung, and the resulting fluid was centrifuged for 8 min at 400×*g* to separate cellular components (cell pellet) from noncellular components (supernatant). The supernatant was collected for protein concentration determination and cytokine ELISA evaluation. The phenotype of the cell pellet was analyzed using flow cytometry.

### Mouse respiratory functional assay

The plethysmograph used to measure pulmonary function *in vivo* was performed using Buxco small animal whole body plethysmography in the Taiwan Mouse Clinic. Mice were treated with PBS (PBS/PBS), LPS (LPS/PBS), IT-sMSC-exo (LPS/IT-sMSC-exo), or miR-7704 (LPS/miR-7704) before being placed individually into a single cylindrical chamber at 0, 6, and 24 h. The mice were allowed to habituate in the Buxco chamber for 15 min before respiratory data were recorded for 1 h. All respiratory parameters (P_enh_, respiratory rate, TV, MV, PIF, and PEF) were quantified following Buxco’s protocol. P_enh_ formula: P_enh_ = $\left(\frac{PEF}{PIF}\right)\times \left(\frac{Te-Tr}{Tr}\right)$.

### Tissue histopathological staining

For histological analysis, the left lung tissue was fixed with 4% paraformaldehyde for H&E or immunohistochemical (IHC) staining after paraffin embedding. Briefly, 4-μm tissue sections were deparaffinized using EZ prep (Ventana Medical Systems). The slides were incubated with anti-F4/80 (Santa Cruz, sc-377009), anti-CD86 (Santa Cruz, sc-28347), and anti-CD206 (Santa Cruz, sc-376108) using the automated Ventana Benchmark XT (Ventana Medical Systems), and detected using the Ultra view DAB Detection Kit (Ventana Medical Systems) following the manufacturer’s protocol. A microscope (OLYMPUS, BX51) was used to randomly select regions at 200× and 400× magnifications. Staining intensity was quantified using ImageJ software with the IHC toolbox plugin. Lung injury scores were assessed following the guidelines outlined in the American Thoracic Society Workshop Report.^43^

### Cell culture

Human adipose tissue-derived MSCs were purchased from LONZA (Catalog #PT-5006). MSCs were cultured in Iscove’s modified Dulbecco’s medium (IMDM; Sigma-Aldrich) supplemented with 10% fetal bovine serum (ES Cell-Qualified FBS; Thermo Fisher Scientific), 1% penicillin-streptomycin-glutamine (PSG; Thermo Fisher Scientific), and 10 ng/mL fibroblast growth factor 2 (Sigma, SI-F0291) at 37°C and 5% CO_2_. The HEK293T cell lines were purchased from ATCC (CRL-3216) and cultured in low-glucose (LG) DMEM (Sigma-Aldrich) supplemented with 10% FBS (Thermo Fisher Scientific) and 1% PSG (Thermo Fisher Scientific) at 37°C and 5% CO_2_.

### *In vitro* differentiation of MSCs

For adipogenic induction of human MSCs, cells were grown in the previously described medium until optimal confluency and then transferred to adipogenic differentiation medium in IMDM supplemented with 0.5 mM 3-isobutyl-1-methylxanthine (Sigma-Aldrich, #28822-58-4), 1 μM hydrocortisone (Sigma-Aldrich, #50-23-7), 0.1 mM indomethacin (Sigma-Aldrich, #53-86-1), and 10% ES-FBS. The induction medium was replaced every 3 days during the 14 days of osteogenic induction. For human MSC osteogenic induction, cells were maintained in the medium previously described until optimal confluency and then transferred to osteogenic differentiation medium: IMDM supplemented with 0.1 μM dexamethasone, 10 mM beta-glycerol phosphate (Sigma Aldrich, 50020), and 0.2 mM ascorbic acid (Nacalai Tesque, 03420-65). The induction medium was replaced every 3 days during the 14 days of osteogenic induction. For chondrogenic induction, cells were maintained in the previously described medium until optimal confluency and then transferred to chondrogenic differentiation medium (LONZA, PT3003) containing transforming growth factor-β3 (LONZA, PT4124), following the manufacturer’s protocol. Chondrogenic induction was carried out for 28 days, with the induction medium replaced every 3 days.

On day 14, Oil Red O staining was used to assess lipid formation in MSCs. The medium was removed, and the cells were rinsed with PBS, fixed with 4% paraformaldehyde for 20 min, and washed twice with distilled water. The fixed cells were stained with Oil Red O (Sigma Aldrich, O0625). Alkaline phosphate histochemistry was performed on day 14. The cells were fixed with 4% paraformaldehyde for 20 min, and then stained with BCIP/NBT (5-bromo-4-chloro-3-indolyl phosphate/nitro blue tetrazolium, Sigma Aldrich, B1911) for 30 min in the dark. Chondrogenic spheroids were collected on day 28. The cells were washed twice with PBS and fixed in 3.7% paraformaldehyde for 1 h. Chondrogenic differentiation was observed using Alcian blue stain (Alcian Blue Staining Solution, Sigma Aldrich, TMS-010-C).

### Preparation of IT-sMSCs

MSCs were cultured at an appropriate cell density (80% confluency) in a serum-free medium with TNF (20 ng/mL, Sigma Aldrich, SRP3177) and IFN-γ (20 ng/mL, Sigma Aldrich, SRP3058) for 24 h at 37°C and 5% CO_2_.

### Exosome isolation and characterization

MSCs were cultured in the presence or absence of TNF and IFN-γ until confluency was achieved. The culture medium was changed to IMDM supplemented with 10% exosome-depleted FBS and 1% PSG. The CM was then collected after 24 h of incubation. The cell debris was removed by centrifuging at 3,000×*g* for 30 min at 4°C, and then filtering through 0.22-μm polyvinylidene difluoride filters. Exosomes were isolated from MSCs and IT-sMSCs using a kit-based method or ultracentrifugation. For the kit-based method, MSC-CM or IT-sMSC-CM was concentrated using a 3 kDa MWCO Vivaspin concentrator (Cytiva, 28932358), and ExoPEG-CM50 (Biovesicle Inc.) was added according to the manufacturer’s protocols. For ultracentrifugation, the CM was centrifuged at 100,000×*g* for 70 min at 4°C, and the obtained exosome pellet was re-suspended using PBS or TRIzol solution for subsequent RNA isolation. Exosomes were characterized using western blotting, transmission electronic microscopy (high contrast transmission electron microscope, Hitachi HT7700), and nanoparticle tracking analysis (NanoSight, NTA 3.1 Build 3.1.45).

### Macrophage isolation, culture, and characterization

C57BL/6 mice were euthanized through isoflurane inhalation overdose, and BM-derived cells from the femur and tibia were flushed out using PBS. The BM-derived cells were incubated with ACK lysis buffer (Thermo Fisher Scientific, A1049201) for 2 min to remove red blood cells. The cells were obtained and differentiated into BMDMs in LG-DMEM (Thermo Fisher Scientific, 11875093) supplemented with 10% FBS (Thermo Fisher Scientific), 1% PSG (Thermo Fisher Scientific), and 20 ng/mL macrophage colony stimulating factor (PeproTech, 315-02). BMDMs were obtained using the previously described medium, which was replaced once every 3 days. Cytokine induction in BMDMs was performed on day 7 for 24 h. Macrophage polarization was induced by treating cells with LPS (100 ng/mL, Sigma Aldrich, L4516) and IFN-γ (45 ng/mL, PeproTech, 315-05) to differentiate M1 macrophages or IL-4 (10 ng/mL, PeproTech, 214-14) to differentiate M2 macrophages. For MyD88 inhibition in M1 macrophages, MyD88 inhibitor or NC was pre-treated in M0 macrophages on day 7 before M1 induction as previously described (LPS+IFN-γ).

### Luciferase reporter assay

WT or MU MYD88 3ʹUTR mRNA was cloned into the pmirGLO vector (E1330, Promega). HEK 293T cells were co-transfected with MYD88 WT or MU plasmid and miR-7704 mimics (100 nM) or NC for 48 h. The luciferase activity was analyzed by following Dual-Glo luciferase assay system’s protocol (Progema) on multimode microplate readers (TECAN SPARK). Relative luciferase activity was normalized to the Renilla:luciferase luminescence ratio.

### RNA extraction and real-time qPCR

Total RNA from exosomes, cells, and mice lung tissue was extracted using TRIzol (Sigma Aldrich, T9424) following the manufacturer’s protocol. Reverse transcription of RNA to cDNA was conducted using an ABI high-capacity cDNA reverse transcription kit (Thermo Fisher Scientific, 4368813). Briefly, 1,000 ng RNA was mixed with 2 μL 10× RT buffer, 2 μL 10× RT random primer, 0.8 μL dNTP (100 mM), and 1 μL transcription enzyme (50 U/μL); the total volume of the mixture was transcribed under the following conditions: 25°C for 10 min, 37°C for 60 min, 85°C for 5 min, and 4°C incubation. The qPCR was conducted in a total reaction mixture of 10 μL containing 20 ng template cDNA in 3 μL ultrapure distilled water (Invitrogen, #10977-015), 5 μL 2×SYBR Green Master Mix (Bio-Rad iTaq Universal SYBR Green Supermix, 172–5124), and 1 μL 10 μM forward and reverse primers. The total reaction for qPCR was incubated at 95°C for 10 min, followed by 40 cycles at 95°C for 15 s and 60°C for 1 min, and a melt curve stage at 95°C for 1 s, 60°C for 20 min, and 95°C for 1 s. The cycle threshold (Ct) values of the target genes were normalized to those of the internal control GAPDH for both mice and humans (ΔCt). Relative expression was calculated as the difference in the ΔCt of the targeting genes from the control group to the treatment group (ΔΔCt). The relative quantitative value was calculated using Cq (2ˆ(−ΔΔCt)). The primer sequences used are listed in Table S1.

### miRNA sequencing and real-time qPCR

miRNAs from exosomes, cells, and mouse lung tissue were isolated using TRIzol (Invitrogen, Cat 15596018), according to the manufacturer’s instructions. Exosomal RNA samples were selected for small RNA sequencing on Illumina HiSeq 2500 with an OD260/OD230 ratio of 1.8 or greater, an OD260/OD280 ratio of 2.0 or greater, and RNA integrity of 8 or greater. Differential expression analysis of miRNAs between MSCs and IT-sMSCs was analyzed using R (RStudio, 3.6.3; limma package) to identify differentially expressed miRNAs based on a p value of less than 0.05 and a log2 fold-change (log2 FC) of greater than 1. A volcano plot of differentially expressed miRNAs of the two groups was created using GraphPad Prism (version 9.02).

For miRNA real-time qPCR, miRNAs were collected and reverse-transcribed to cDNA using SuperScript III Reverse Transcriptase (Invitrogen, 18080044). In brief, a mixture of 1 μL RNA, 0.5 μL dNTP (10 mM), and 1 μL Stem-loop primer (1 μM) was incubated using Program I: “65°C for 5 min→4°C for at least 2 min.” The product was then mixed with 4 μL 5× First-Strand buffer, 2 μL DTT, 0.1 μL RNaseOUT (Invitrogen, 10777019), and 0.25 μL SuperScript III Reverse Transcriptase and incubated using Program II: “16°C for 30 min→60 cycles of 30°C for 30 s→42°C for 30 s→50°C for 1 s→85°C for 5 min.” The primer sequence list is presented in Table S1. The total volume for miRNA real-time qPCR was 20 μL, containing 2 μL template cDNA in nuclease-free water, 4 μL TaqMan Master Mix (Thermo Fisher Scientific, #4304437), 1 μL 10 μM primers (forward and reverse), and 0.2 μL 10 μM Universal Probe #21 (Roche, 4687612001) incubated using Program III: “95°C for 5 min→45 cycles of 95°C for 5 s→60°C 10 s→72°C for 1 s.” The primer sequences are listed in Tables S3 and S4. miRNA sequencing and region in detail were gained from the Ensembl Project database (https://asia.ensembl.org/index.html).^50^

### miRNA transfection to the cells

Mouse BMDMs were seeded into a 6 cm dish at a density of 1 × 10^6^ cells/well, and miRNA transfection was performed using TOOLSmartFect Transfection Reagent (TN-S01, BIOTOOLS Co., Ltd.), according to the manufacturer’s protocol, with an miR-7704 mimic (100 nM), anti-miR-7704 (100 nM), or their NC (100 nM) (Table S6) (BIOTOOLS Co., Ltd.). The miR-7704 mimic conjugated with Cy5 immunofluorescence or inhibitor and the transfection reagent were mixed with basal LG-DMEM medium (Thermo Fisher Scientific, 11875093) without FBS as described previously, and the transfection complex was formed after 30 min of incubation. The miRNA complexes were transfected into cells for 48 h, and transfection efficiency and efficacy were determined using real-time qPCR, flow cytometry, ELISA, and western blotting.

### Proteomics analysis

The proteins extracted from the macrophages were analyzed using LC-MS/MS (Orbitrap Elite; Thermo Fisher Scientific). Data analysis was conducted using Proteome Discoverer software (version 1.4, Thermo Fisher Scientific), including the reporter ion quantifier node for iTRAQ quantification. The MS/MS spectra were searched under the NCBI (RefSeq) database. The following parameters were used for peptide identification: 15 ppm of precursor mass tolerance and 0.5 Da for CID/0.2 Da for HCD fragment ions, allowing for two missed cleavages. The peptide-spectrum match was then carried out using high confidence and Mascot search engine rank 1 for peptide identification to ensure an overall false discovery rate of less than 0.01. The protein ratios in each group were normalized to the median of the control groups.

### Western blotting

The exosomes and mouse lung tissue were lysed and quantified. Briefly, 20 μg protein was separated on a 10% polyacrylamide gel (BioShop, ACR009.500) and transferred to a polyvinylidene difluoride blotting membrane. The membrane was blocked using 5% BSA in Tris-buffered saline and 0.1% Tween 20 for 1 h and probed with antibodies (CD9, Invitrogen, #10626D; CD63, Novus Biologicals; Alix, Cell Signaling, #92880; STAT1, Cell Signaling, #66545-1-Ig; pSTAT1, Cell Signaling, #9167S; MyD88, Cell Signaling, #4283) at 4°C overnight. The membrane was incubated with horseradish peroxidase (HRP)-conjugated secondary antibodies for 2 h. Protein levels were detected using TOOLS Ultra ECL-HRP substrate (TOOLS, #TU-ECL02, BIOTOOLS Co., Ltd.). Chemiluminescence was imaged using the Cytiva Imager 680 system (Cytiva, #29270769).

### Flow cytometry

For flow cytometry analysis, 1 × 10^6^ cells were harvested, washed with flow cytometry staining buffer (2 mM EDTA with 0.5% BSA in PBS), and stained with the following antibodies. MSCs were stained with CD90 (BD Pharmingen, PE, Cat. 561970), CD105 (eBioscience, PE, Cat.12-1057-41), CD73 (eBioscience, FITC, Cat.11-0739-41), CD45 (BD Pharmingen, FOTC, Cat.560976), CD14 (eBioscience, FITC, Cat.11-0149-73), CD34 (Invitrogen, FITC, 11-0349-42), CD19 (Invitrogen, PE, 12-0199-42), and HLA-DR (Invitrogen, PE, 12-9956-42). Mouse macrophages or BALF-derived cells were stained with CD11b (BD Pharmingen, APC, #5553312), CD86 (BD Pharmingen, FITC, Cat. 553691), and CD206 (BD Pharmingen, Alex Fluor 647, Cat. 565250).

### ELISA

Cell culture CM or BALF from mice were collected and analyzed using the Multiplex ELISA Kit for Mouse Cytokine Panel 1 (6-Plex, MEK1011, BOSTER) following the manufacturer’s instructions. All samples were diluted at a 1:2 ratio, and the results were analyzed using Q-View software (Quansys Biosciences).

### Statistical analysis

Statistical data were processed using GraphPad Prism (GraphPad Software, Version 9.02) as mean ± SD with one-way ANOVA, two-way ANOVA, and unpaired Student’s t test, depending on experimental design. Significance was considered at a p value of less than 0.05. Figure 1A and the graphical abstract were created with templates and materials from BioRender.com.

## Data and code availability

The datasets used and/or analyzed during the current study are available from the corresponding author on reasonable request.

## Supplemental information

Document S1. Figures S1–S4 and Tables S1–S6

Document S2. Article plus supplemental information
