# Supplementary material for: Modulation of experimental acute lung injury by exosomal miR-7704 from mesenchymal stromal cells acts through M2 macrophage polarization
Source: Mol Ther Nucleic Acids. 2023 Dec 14;35(1):102102. doi: 10.1016/j.omtn.2023.102102 (PMC10787251; doi:10.1016/j.omtn.2023.102102)
Supplement: Document S1. Figures S1–S4 and Tables S1–S6 [file mmc1.pdf]

OMTN, Volume *35*

Supplemental information

Modulation of experimental acute lung injury by exosomal miR-7704 from mesenchymal stromal cells acts through M2 macrophage polarization

Wei-Ting Lin, Hao-Hsiang Wu, Chien-Wei Lee, Yu-Fan Chen, Lawrence Huang, Jennifer Hui-Chun Ho, and Oscar Kuang-Sheng Lee

Supplemental material

Figure S1

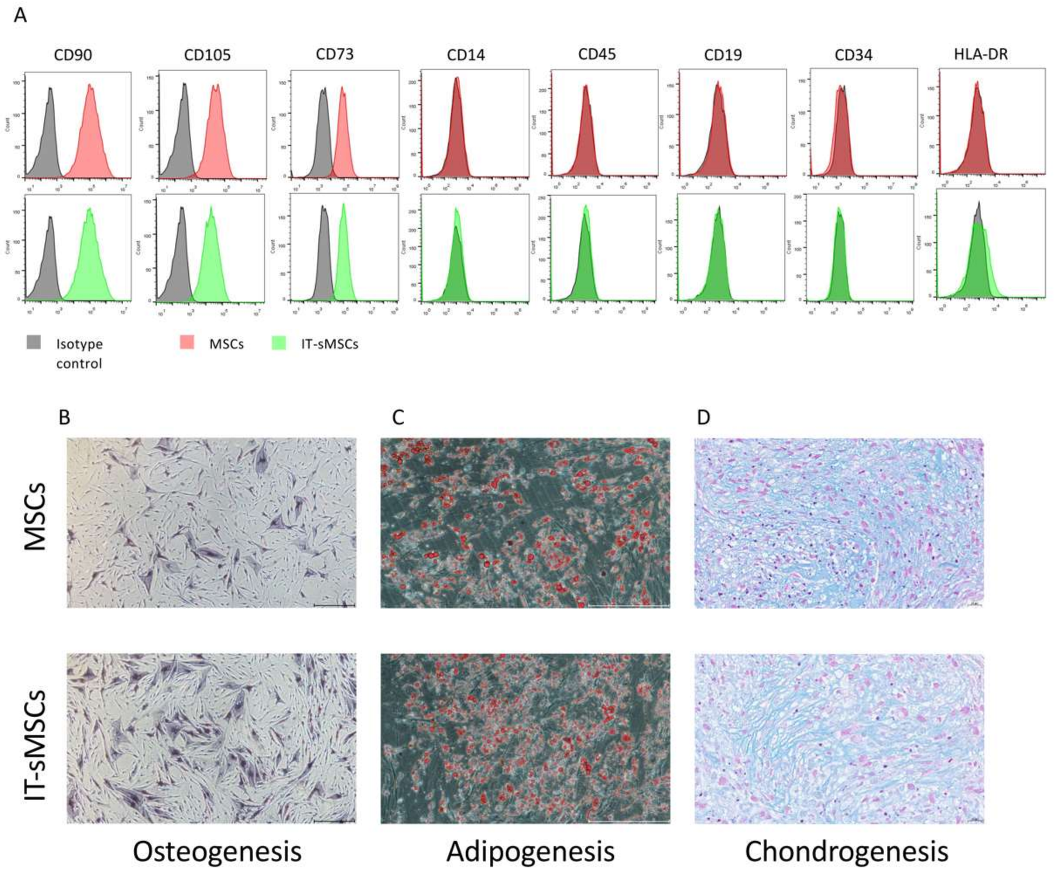

Figure S1. Characterization and tri-lineage differentiation of MSCs and IT-sMSCs.

(A) MSCs and IT-sMSCs surface markers were determined through flow cytometry (each group n = 6). Graphic showed tri-lineage differentiation of MSCs (upper panel) and IT-sMSCs (downer panel). (B) Osteogenic differentiation was characterized using ALP staining (scale bar = 200 µm. (C) Adipogenic differentiation was confirmed using Oil red

O staining (scale bar = 100 µm. (D) Chondrogenic differentiation was measured using Alcian blue staining (scale bar = 200 µm).

Figure S2

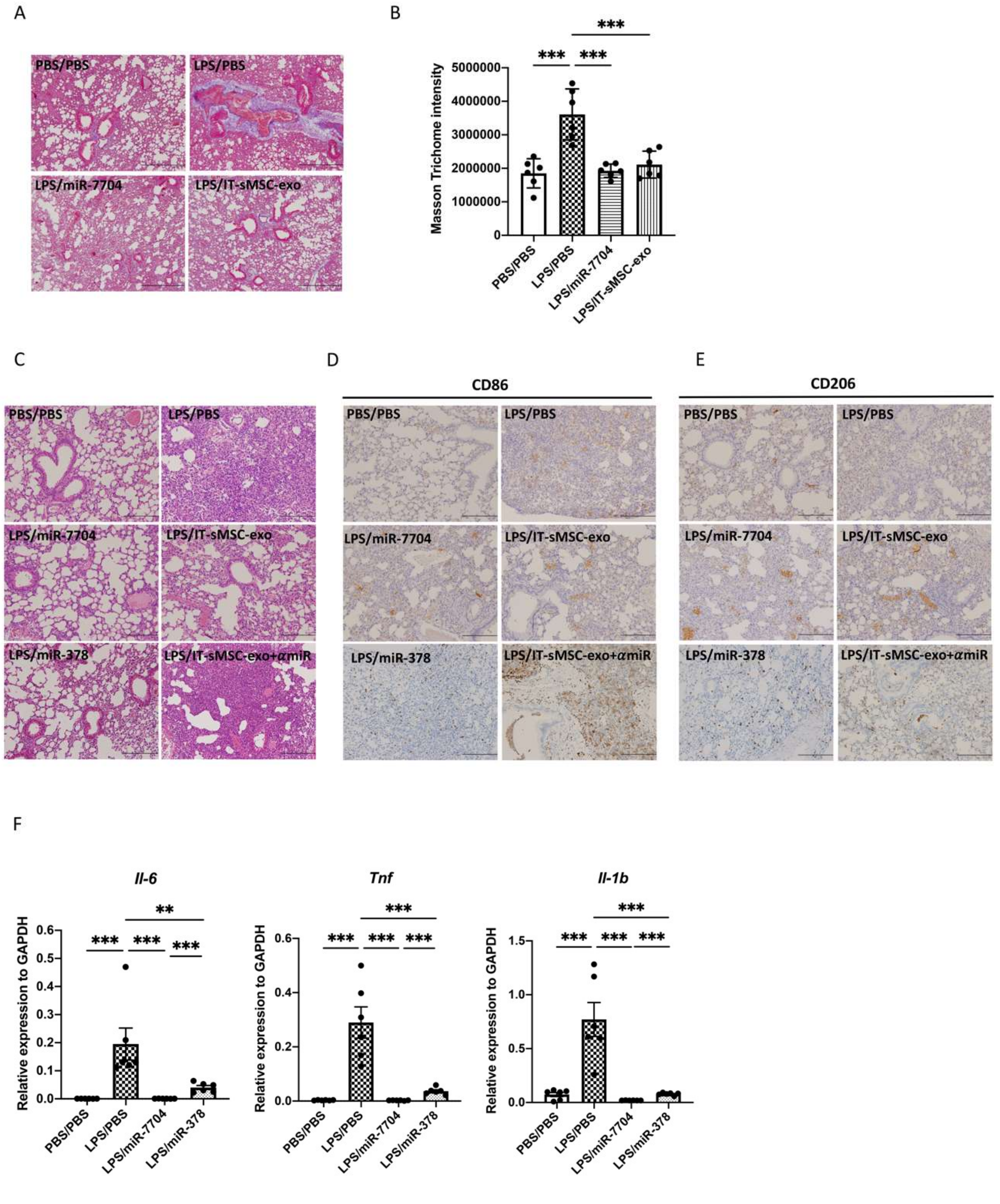

Figure S2. Effects of miR-378 and anti-miR-7704 in ALI mouse model

(A) Representative Masson trichome staining and staining intensity (B) of lung tissue in ALI mouse model (scale bar = 500 μm). (C) H&E staining of lung tissue in miR-378 groups (LPS/miR-378) and IT-sMSC-exo plus anti-miR-7704 groups (LPS/IT-sMSC-exo-αmiR). (D) CD86 and (E) CD206 IHC staining in miR-378 groups (LPS/miR-378) and IT-sMSC-exo plus anti-miR-7704 groups (LPS/IT-sMSC-exo-αmiR). (F) Gene expression of *IL-6*, *TNF*, and *IL-1β* in lung tissue of ALI mouse model. Results are presented as mean ± SD. Statistical analyses were performed using one-way ANOVA (*p < 0.05, **p < 0.01, ***p < 0.001; ns, not significant).

Figure S3

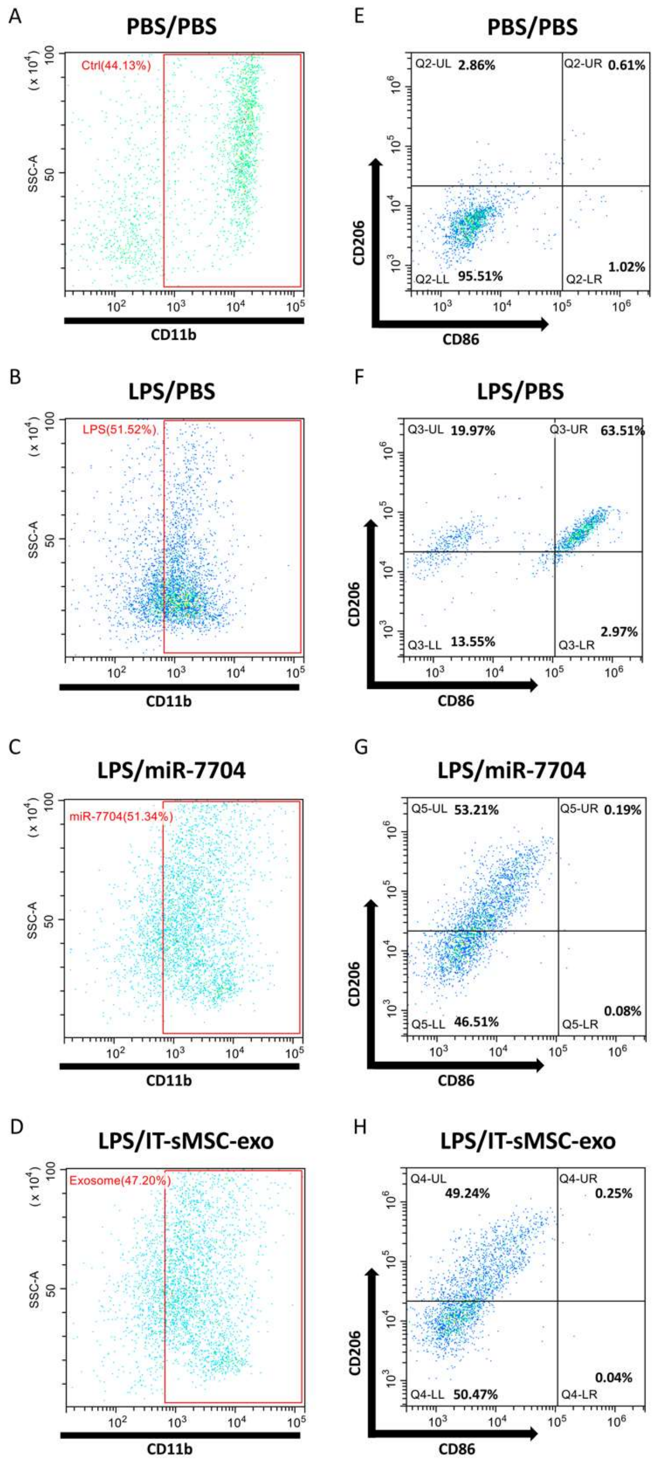

Figure S3. Characterization of BALF-derived cell in LPS-induced ALI mice

BALF-derived cell phenotypes were analyzed using flow cytometry. (A–D) CD11b-positive cells in different groups. (E–H) CD86 and CD206 expression in different groups.

Figure S4

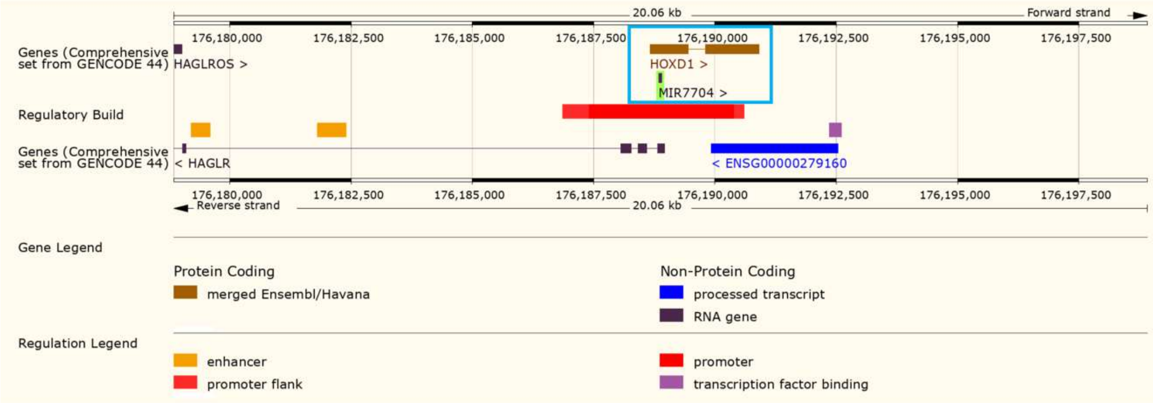

Figure S4. Region in detail of miR-7704 and HOXD1 gene in chromosome 2

Tables

Table S1

The list of primer sequences used for qPCR

Target	Sequence
GAPDH	Forward: GTCTCCTCTGACTTCAACAGCG<br>Reverse: ACCACCCTGTTGCTGTAGCCAA
IDO	Forward: GTGTTTCACCAAATCCACGA<br>Reverse: CTGATAGCTGGGGGTTGC
PGE2	Forward: GGATCTGTGGATGCTTCGTT<br>Reverse: ACCCACAGTGCTTGACACAG
CXCL10	Forward: GAAAGCAGTTAGCAAGGAAAGGT<br>Reverse: GACATATACTCCATGTAGGGAAGTGA
CXCL9	Forward: TGTTCCCCTTTGCTTCATTC<br>Reverse: GAAAGGCACTGCATTGTGG
Gapdh	Forward: GGGAAGCCCATCACCATCT<br>Reverse: CGGCCTCACCCCATTTG
Il-6	Forward: ACAAAGCCAGAGTCCTTCAGA<br>Reverse: ACAAAGCCAGAGTCCTTCAGA
Tnf-α	Forward: CTGTAGCCCACGTCGTAGC<br>Reverse: CTGTAGCCCACGTCGTAGC
Ifn-γ	Forward: ATCTGGAGGAACTGGCAAAA<br>Reverse: TTCAAGACTTCAAAGAGTCTGAGG
Inos	Forward: CTTTGCCACGGACGAGAC<br>Reverse: CTTTGCCACGGACGAGAC
Il-1β	Forward: AGTTGACGGACCCCAAAAG<br>Reverse: AGTTGACGGACCCCAAAAG
IL-18	Forward: CAAACCTTCCAAATCACTTCCT<br>Reverse: TCCTTGAAGTTGACGCAAGA
Cd80	Forward: TTCGTCTTTCACAAGTGTCTTCA<br>Reverse: TGCCAGTAGATTCGGTCTTCA
Cd86	Forward: GAAGCCGAATCAGCCTAGC<br>Reverse: CAGCGTTACTATCCCGCTCT
Cd163	Forward: TCACTTCTCAGTGCCTCTGC

	Reverse: CGCCAGTCTCAGTTCCTTCT
Cd206	Forward: CCACAGCATTGAGGAGTTTG
	Reverse: CCACAGCATTGAGGAGTTTG
Arg1	Forward: GAATCTGCATGGGCAACC
	Reverse: GAATCCTGGTACATCTGGGAAC
Il-10	Forward: CAGAGCCACATGCTCCTAGA
	Reverse: CAGAGCCACATGCTCCTAGA

Table S2

Top 10 enriched miRNAs in IT-sMSC-exo

**Identifier**	**IT-sMSC-exo DE**	**MSC-exo DE**	**log2Fold change**	**p value**
hsa-miR-7704	1204.19562	143.305936	3.070899506	0.0010103
hsa-miR-4516	53.7804425	9.33621785	2.526171496	0.0309224
hsa-miR-378b	1451.25074	327.822371	2.146310591	0.0020834
hsa-miR-378e	1457.83595	338.307479	2.107421401	0.002292
hsa-miR-378f	3650.19081	960.83486	1.925611483	0.0030204
hsa-miR-378g	3675.34567	970.034689	1.921771698	0.0031302
hsa-miR-378d	3773.63731	1015.41679	1.893883756	0.0034252
hsa-miR-378h	523.895416	145.350703	1.849740793	0.0022665
hsa-miR-27a-5p	83.7341512	27.0646775	1.62940495	0.0112302
hsa-miR-378i	16534.027	5370.1239	1.622410865	0.0172634

Table S3

miRNA reverse transcriptase stem-loop primer list

Target	Sequence
miR-7704	GTTGGCTCTGGTGCAGGGTCCGAGGTATTCGCACCAGAGCCAACCACGTC
miR-378	GTTGGCTCTGGTGCAGGGTCCGAGGTATTCGCACCAGAGCCAACTTCTGC

Table S4

miRNA qPCR primer list

Target	Sequence
miR-7704	CGGGGTCGGCGGCGAC
miR-378	GGACTGGACTTGGAGGCA
Universal reverse primer	GTGCAGGGTCCGAGGT

Table S5

GO enrichment of proteomic analysis

Trend	Term	Count	Genes	P Value	FDR
down-regulation	GO:0035458~cellular response to interferon-beta	5	TGTP2, STAT1, IFI47, GBP2, IIGP1	4.5E-06	0.00199
down-regulation	GO:0071346~cellular response to interferon-gamma	4	GBP7, NOS2, GBP2, GBP4	0.00083	0.0919
down-regulation	GO:0071222~cellular response to lipopolysaccharide	4	NOS2, STAT1, CD14, GBP2	0.019	0.49
down-regulation	GO:0002376~immune system process	5	FCGR1, DBNL, PRKCB, CD14, IIGP1	0.0199	0.49
down-regulation	GO:0031663~lipopolysaccharide-mediated signaling pathway	2	STAT1, CD14	0.0807	0.993
down-regulation	GO:0071345~cellular response to cytokine stimulus	2	NOS2, STAT1	0.0857	0.993
down-regulation	GO:0032496~response to lipopolysaccharide	3	NOS2, STAT1, SOD2	0.0996	0.993
down-regulation	GO:0055114~oxidation-reduction process	7	NOS2, UQCRC1, OGDH, HMOX1, SOD2, HSD17B11, HSD17B10	0.00952	0.423

down-regulation	GO:0042542~response to hydrogen peroxide	3	STAT1, HMOX1, SOD2	0.0108	0.436
down-regulation	GO:0001666~response to hypoxia	4	NOS2, PRKCB, HMOX1, SOD2	0.0152	0.49
down-regulation	GO:0051770~positive regulation of nitric-oxide synthase biosynthetic process	2	STAT1, NAMPT	0.0477	0.933
down-regulation	GO:0006801~superoxide metabolic process	2	NOS2, SOD2	0.0503	0.933
down-regulation	GO:0006979~response to oxidative stress	3	HMOX1, SOD2, GCLM	0.0504	0.933
down-regulation	GO:0006096~glycolytic process	2	PFKL, OGDH	0.0932	0.993

Table S6

The list of miRNA mimic, inhibitor and negative control sequences

product	Sequence (5'→3')
miR-7704 mimic	CGGGGUCGGCGGCGACGUG<br>CGUCGCCGCCGACCCCGUU
miR-7704 inhibitor	CACGUCCCCCGACCCCG
miR-378 mimic	ACUGGACUUGGAGGCAGAA<br>CUGCCUCCAAGUCCAGUUU
Negative control	UUC UCC GAA CGU GUC AUG UTT<br>ACG UGA CAC GUU CGG AGA ATT
